# Guns, violence, politics: the gyre widens

**DOI:** 10.1186/s40621-021-00357-3

**Published:** 2021-11-02

**Authors:** Garen J. Wintemute

**Affiliations:** Department of Emergency Medicine, California Firearm Violence Research Center, University of California, Davis, USA

**Keywords:** Firearms, Violence, Community violence, Political violence, Domestic terrorism

## Abstract

Inter-related sustained upward trends in firearm purchasing, violence, and political extremism are converging to put the USA at risk for disaster and threaten our future as a democracy. This narrative review provides a critical assessment and call to action. It explores each trend separately, considers the effects of their likely and imminent convergence, and suggests possibilities for collective and individual action to prevent or at least reduce those effects.

Inter-related upward trends in firearm purchasing, violence, and political extremism are converging to put the USA at risk for disaster in the months ahead. We have no time to waste if we are to prevent the loss of thousands of lives and emerge with our democracy intact.

## Firearm purchasing

**Fig. 1:**
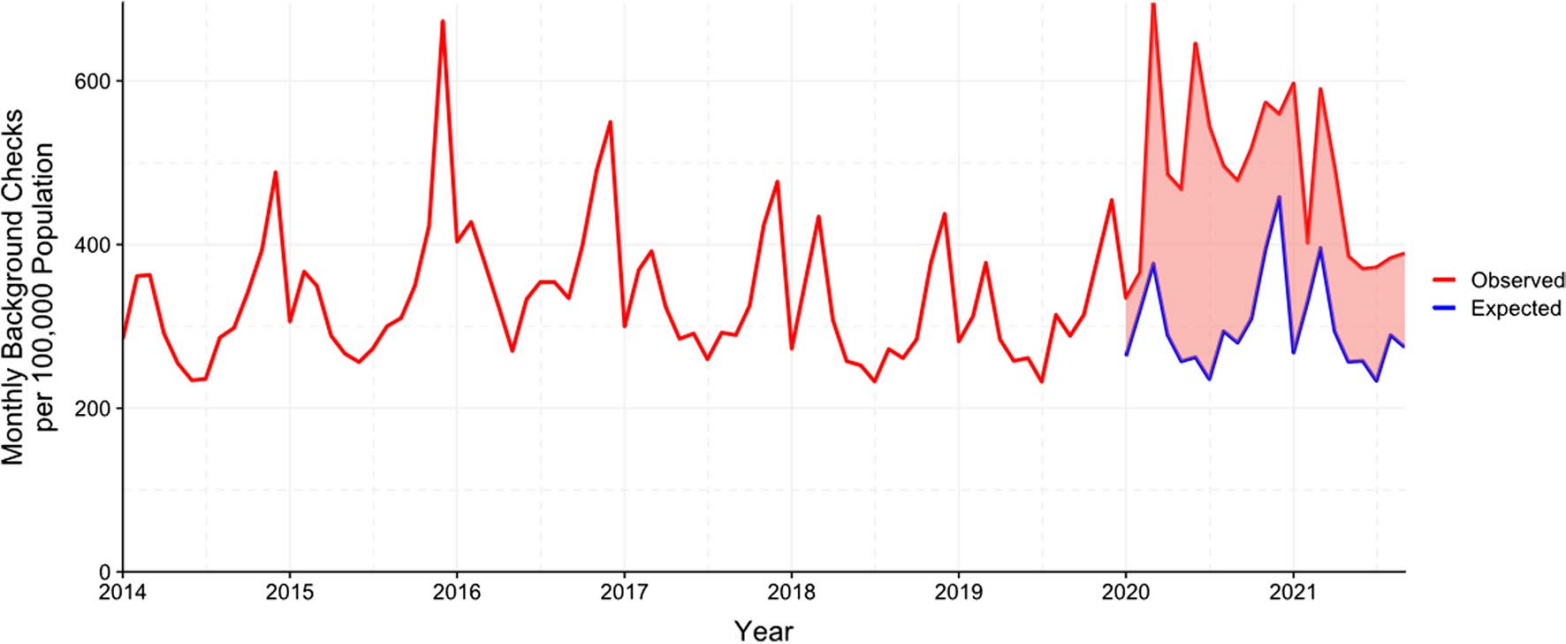
Monthly Background Checks for Firearm Purchases, January 2014 through September 2021. Checks are conducted by the Federal Bureau of Investigation’s National Instant Criminal Background Check System. Counts of observed background checks were obtained at https://www.fbi.gov/file-repository/nics_firearm_checks_month_year_by_state_type.pdf/view

A pandemic loomed in January 2020, and by March it had arrived; there was the prospect and then the reality of widespread social disruption. People purchase firearms more for protection than for all other reasons combined (Azrael et al. 2017; Igielnik and Brown 2017), and background checks for firearm purchases increased early in the year to well beyond expected levels (Fig. 1; expected counts for 2020–2021 were obtained by fitting an ARIMA model to data for January 2014 through December 2019).

Then came spring, George Floyd’s murder, and perhaps unprecedented recognition and intense debate over the structural injustices that facilitate and perpetuate violence. There were additional instances of police violence and reactions to them, a decline in the perceived legitimacy of the police, and reports of increases in violent crime in major cities and in hate crimes everywhere. Excess firearm purchasing continued through the summer.

Fall 2020 brought presidential election season—always a stimulus to buy firearms—and this time the gun control candidate won. With possibility of more restrictive firearm policies in the air, the purchasing surge continued past the election. Then, President Trump and his followers denied his loss. Political instability followed; in January 2021, telling his supporters to “fight like hell” (Byman 2021), Trump incited a violent assault on the Capitol. Through the first 9 months of 2021, as the COVID-19 pandemic and political and social instability remained dominant features of the American landscape, the purchasing surge continued (Fig. 1).

Today, we remain in an unprecedented surge in firearm purchasing that shows no sign of abating and risks becoming part of a new normal for the USA. Background checks on firearm purchasers for January 2020 to September 2021 are 59.9 % above expected levels (Fig. 1). An estimated 12.5 million excess background checks have been conducted, of 33.4 million checks altogether. According to FBI data going back more than 20 years, 6 of the 10 days, and 6 of the 10 weeks, with the highest number of background checks for firearm purchases have occurred this year (Federal Bureau of Investigation 2021a).

Bear in mind that background checks underestimate actual purchases. They do not account for purchases of multiple firearms in a single transaction. They do not include all purchases from private parties; about 20% of firearm transactions do not involve background checks (Miller et al. 2017). They do not include privately manufactured “ghost guns” (Wintemute 2021).

Four independent surveys have been conducted of firearm purchasers during the pandemic (Kravitz-Wirtz et al. 2021; Azrael and Miller 2021; Tavernise 2021; Crifasi et al. 2021; Sokol et al. 2021). The findings published so far suggest that the population of gun owners is evolving rapidly. Recent purchasers are younger than others and less likely to be male and non-Hispanic white; at least 20% are first-time buyers.

## Violence

By spring 2020, violence was also increasing beyond expected levels. Homicide, the violent crime most likely to involve firearms, rose by nearly 27.5 % nationwide in 2020 (Federal Bureau of Investigation 2020)—a year-over-year relative increase far greater than anything seen in the past 100 years (https://www.cdc.gov/injury/wisqars; Justice Research and Statistics Association 2000)—and by more than 30% in many major cities (Rosenfeld et al. 2021). In the first half of 2021, homicide rates in major cities increased another 16%; the January–June homicide rate for 2021 was 42% above that for 2019 (Rosenfeld and Lopez 2021). (Not all violent crimes exhibited large increases in 2020. Aggravated assault rose 11.7% from 2019 to 2020, ending a decade of little change; robbery, continuing a long-term downward trend, fell 9.7% (Federal Bureau of Investigation 2020)).

Hate crimes, which numbered about 300,000 in 2019 according to the National Crime Victimization Survey (Kena and Thompson 2021), increased 6.5% in 2020 (the 2020 figure is for offenses reported to law enforcement and is subject to substantial under-reporting) (https://crime-dataexplorer.app.cloud.gov/pages/explorer/crime/crime-trend). In California, reported hate crimes rose 31% overall in 2020 and 67% for crimes involving racial bias (Criminal Justice Statistics Center, California Department of Justice 2020). In the first quarter of 2021, police-reported anti-Asian American hate crimes in 16 large cities and counties nationwide rose 164%, compared with the first quarter of 2020 (Levin 2021). Anecdotal reports suggest that the changing demographics of firearm purchasers partly represent purchasing by members of groups that are targeted by hate crime perpetrators (Fisher et al. 2021).

A study of trends from January through May 2020 found a strong state-level association between the size of the increase in firearm purchasing and the size of the subsequent increase in firearm violence (Schleimer et al. 2020). By July, however, as violence increased in response to seasonal trends, social unrest, and other factors, that association was no longer detectable for firearm violence overall, but persisted for intimate partner violence (Schleimer et al. 2021).

## Politics

Recent public opinion polls paint a very grim portrait of alienation and readiness for political violence in the USA. Nearly 70% of adults, with very similar results for Democrats and Republicans, agree that “American democracy only serves the interests of the wealthy and powerful” (Cox 2021); fewer than 20% believe that political leaders will “do what is right for [me] and [my] community” (Public Religion Research Institute/Interfaith Youth Corps 2021). Approximately 20% of Republicans, conservatives, and voters for Donald Trump take this position to its extreme and disagree with the proposition that “democracy is [the] best form of government” (The Economist/YouGov 2021).

Many Americans have entered a world of paranoid delusion—a world that exists because, as sociologist Zeynep Tufekci describes it, “belonging is stronger than facts” (Tufekci 2018). Nearly 30% of adults, and nearly two-thirds of Republicans, believe that President Biden was not elected legitimately (Cox 2021; Public Religion Research Institute/Interfaith Youth Corps 2021). One in 6 adults subscribes to the QAnon myth that “the government, media, and financial worlds in the USA are controlled by a group of Satan-worshiping pedophiles who run a global child sex trafficking operation” (Public Religion Research Institute 2021). One in 5 believes that “there is a storm coming soon that will sweep away the elites in power and restore the rightful leaders”; 1 in 3 sees conditions in the country as “evidence that we are living in what the Bible calls ‘the end times’” (Public Religion Research Institute 2021).

Support for political violence is no longer only an extremist’s position. More than a third (36%) of American adults and 56% of Republicans agree that “the traditional American way of life is disappearing so fast that we may have to use force to save it” (Cox 2021). One person in 6 agrees that “because things have gotten so far off track, true American patriots may have to resort to violence in order to save our country” (Public Religion Research Institute 2021). Nearly two-thirds (65%) of Republicans, 70% of independents, and 78% of Democrats expect elections to lead to violence (Agiesta 2021).

Policymakers have fed the fire: endorsing a military coup (Astor 2021; Blake 2021) and executions of FBI agents and elected officials (“the only way you get your freedoms back is it’s earned with the price of blood” (Follman 2021)), denying the nature of January’s insurrectionist assault on the Capitol (McManus 2021), planning and facilitating a similar assault on the Oregon State Capitol in Salem (Baker 2021a).

Now, recently enacted policies and others still being debated are poised to increase both the likelihood and lethality of political violence. A wave of voter suppression legislation reminiscent of Jim Crow is sweeping conservative states. As of October, 19 states had enacted 33 laws restricting access to voting (Brennan Center for Justice 2021). Echoing President Biden, who called such legislation an “assault on democracy” (Biden 2021), nearly 200 experts concluded that “our entire democracy is now at risk” (Statement of concern 2021).

Many of those states and others are simultaneously loosening restrictions on firearms. Some are enacting laws allowing the carrying of firearms in public without a permit, which are in effect in at least 20 states (Thrush and Bogel-Burroughs 2021). At least 9 states have acted to interfere with the enforcement of federal gun statutes (Thrush and Bogel-Burroughs 2021).

Law enforcement experts also speak of assault on democracy, but they mean it literally. In June, the FBI predicted that “the current environment likely will continue to act as a catalyst for some…adherents of QAnon … towards engaging in real world violence” (Federal Bureau of Investigation 2021b). The National Security Council reached an even bleaker conclusion in March, predicting that “[n]ewer sociopolitical developments … will almost certainly spur some DVEs [domestic violent extremists] to try to engage in violence this year” (National Security Council 2021). One expert put it this way: “A lot of people want to see January 6 as the end of something. I think we have to consider the possibility that this was the beginning of something” (Taub and Bennhold 2021).

## Convergence

Decades of research at the population and individual levels have established that increased prevalence of firearm ownership and access to firearms are associated with increases in interpersonal violence and self-harm. The USA is still experiencing an unprecedented surge in firearm purchasing and has no choice but to live through its effects. Our challenge is to do everything we can to limit the harm that will almost surely follow.

This would be difficult under any circumstances, but today, to quote my colleague Professor Shani Buggs, “what we have is compounded trauma. The pandemic exacerbated all of the inequities we had in our country—along racial lines, health lines, social lines, economic lines. All of the drivers of gun violence pre-pandemic were just worsened last year” (Thebault et al. 2021).

We also face the real prospect of large-scale political violence. If that still sounds like unrealistic pessimism, consider what will happen next year when armed voter suppression (surely that’s coming) meets armed voter support. Perhaps vaccine or mask mandates will trigger more than isolated outbreaks of violence. Or perhaps the flashpoint will be a more focused conflict, such as private enforcement of an abortion ban in Texas or the fight over water rights in the ever-hotter and -dryer West. Of the latter possibility, Ammon Bundy—son of Cliven, candidate for governor of Idaho, and past user of armed force to seize public resources—said this: “Who cares if there is violence? At least something will be worked out” (Baker 2021b).

Let me make a case for optimism, or at least for hope. Crises are opportunities. The country has a broader and deeper awareness of the structural causes of violence than at any time in the past 50 years, if not longer. In a July 2020 survey on approaches to crime prevention, “increased funding for economic opportunities in poor communities” was the most widely supported strategy among American adults, endorsed by 75% overall and by strong majorities across the political spectrum (Wootson and Clement 2021). Support for “using social workers to help police defuse volatile situations” (65%) exceeded that for “more funding for police departments” (55%).

The Biden administration is proposing to spend $5 billion over 8 years to support evidence-based community violence prevention programs and billions more for workforce development in underserved communities (The White House 2021a). The first-listed priority of the Department of Justice’s violent crime reduction program is to “build trust and earn legitimacy” (Department of Justice 2021), which are at very low levels (Page and Lee 2021). A new $100 million initiative targets domestic violent extremism, including its causes and accelerators (The White House 2021b). The Bureau of Alcohol, Tobacco, Firearms and Explosives is poised to undertake a rigorous, data-based approach to interrupting the supply of firearms for criminal use. If adopted, these large-scale initiatives could provide the opportunity for fundamental change to a comprehensive approach to violence that is not executed primarily by people with guns.

As of late October 2021, however, it is by no means certain that these important initiatives will come to pass. Even if they do, government—especially good government—can only do so much. Recapturing the future of the USA is our responsibility, yours and mine, individually and together.

Under similar circumstances a century ago, Irish poet William Butler Yeats observed in “The Second Coming” that “things fall apart; the center cannot hold; /… The best lack all conviction, while the worst/are full of passionate intensity.” For things not to fall apart this time, we must act on our conviction that the structures that engender and perpetuate violence were built purposefully and must be taken down just as purposefully.

We all come equipped with tools for the job. Each of us can publicly reject violence, including political violence, and say something if we see something. Each of us can work to reduce disparity, increase opportunity, and build inclusive communities that will bring to life the possibilities shining out from the 2020 Census. Each of us can support initiatives to improve education; income, housing and job security; law enforcement and criminal justice policies and practices; and access to medical and mental health care. Each of us can help make sure that all of us can vote, which is how democracy finds its voice.

I have a specific suggestion on how to proceed. Begin by answering the following questions: Of the issues I’ve mentioned and others, which few rise to the top for you? For which of those few are you best equipped to effect change? Then make a task list—your objectives should be specific, measurable, and achievable—and give it a timeline. Tell others what you have set out to do, keep them apprised of your progress, and ask for their help if appropriate. Evaluate and learn from your actions; add new tasks as you complete those you begin with.

## Conclusions

Optimism can come hard at times like this. But the proper reaction to the threat of violence is not more violence—nor is it giving up. Instead, as others do, I argue that we have no choice but to act now as if fundamental change is possible, doing the best we can with what we have. That’s all we’ve ever been able to do.

## Data Availability

Not applicable.
